# Urban Water Leakage Detection System over Dark Fiber Networks Based on Distributed Acoustic Sensing and Sparse Autoencoders

**DOI:** 10.3390/s26072152

**Published:** 2026-03-31

**Authors:** Vahid Sharif, Yuanyuan Yao, Alayn Loayssa, Mikel Sagues

**Affiliations:** 1 Institute of Smart Cities, Department of Electrical, Electronic and Communications Engineering, Universidad Pública de Navarra, Campus de Arrosadia, 31006 Pamplona, Spain; vahid.sharif@unavarra.es (V.S.); alayn.loayssa@unavarra.es (A.L.); 2 College of Engineering and Applied Sciences, Nanjing University, Nanjing 210023, China; dg20340012@smail.nju.edu.cn

**Keywords:** distributed acoustic sensing, optical pulse compression, dark fiber, urban water networks, leakage detection, anomaly detection

## Abstract

We propose and experimentally validate an automatic urban water leakage detection architecture that leverages dark fiber links already deployed in telecommunication networks in underground conduits in the vicinity of water pipelines. The sensing stage relies on a differential-phase coherent optical time-domain reflectometry interrogator enhanced with optical pulse compression to improve sensitivity. Building on this vibration acquisition stage, automatic leakage detection algorithms are implemented by searching for leak-induced activity in the frequency domain, which is well suited to revealing leakage-related features. After acquiring a baseline calibration to characterize normal-condition vibrations at each sensing position, leakage candidates are identified by comparing distribution-based metrics computed over multiple measurements against the corresponding baseline statistics. Two automatic leakage detection strategies are developed. First, low-complexity feature-based metrics are implemented, enabling continuous monitoring with minimal computational requirements. Second, an autoencoder-based anomaly detection technique is introduced, which also relies on location-specific normal-condition calibration but reduces the dependence on prior knowledge of the expected leakage vibration signatures. A real-world field trial on an urban network demonstrates reliable detection and localization using controlled leak events generated in the field, with measurements performed over a 17 km sensing fiber and an effective spatial resolution of 2.6 m. Benchmarking against a commercial punctual electro-acoustic leak detector yields consistent trends. Overall, the proposed system could complement existing technologies by enabling automated, continuous city-scale monitoring over already deployed dark fiber infrastructure.

## 1. Introduction

Non-revenue water has long posed significant economic and environmental challenges for governments, primarily due to leaks in supply pipelines [1,2]. According to World Bank estimates, approximately 15% of the water supplied through distribution networks is lost due to pipeline leakage in developed countries, whereas losses can reach about 30% in developing countries [3]. Leak occurrence is strongly influenced by operating conditions and the progressive deterioration of assets. In particular, buried pipes experience external loads, soil–pipe interactions, and material aging, which increase the likelihood of cracks and joint failures over time [2,4]. As a result, a wide variety of leak detection technologies have been proposed, which can be broadly grouped into two categories. On the one hand, hydraulic methods (e.g., flow meter and pressure analytics) are mainly used to indicate the presence of leaks within a given pipe or distribution sector [5,6]. On the other hand, vibration-based approaches rely on the fact that leaks generate detectable vibro-acoustic emissions that propagate along the pipelines and can be exploited for both leakage detection and localization. The latter are particularly suitable for drinking water distribution networks, which are typically pressurized [7,8]. However, there is a key operational gap between detecting that a leak exists and localizing it with actionable accuracy. While district-level indicators (including flow meter imbalances) can flag abnormal losses, the precise leak position often still requires labor-intensive inspection along the network, which becomes costly and difficult to scale in dense urban deployments [7]. In fact, in-field acoustic surveys performed by trained technicians are still widely used in practice. These surveys rely on punctual electro-acoustic sensors that are manually coupled to the pavement surface or, when accessible, placed in direct contact with the pipeline inside valve chambers.

Consequently, continuous monitoring of large-scale pipeline networks remains challenging, particularly when utilities seek wide-area coverage with actionable leak localization. This challenge has prompted several research groups to explore scalable sensing solutions based on distributed fiber optic technologies. In this context, distributed fiber optic sensing is attractive because a single cable can provide spatially resolved measurements over many kilometers. Moreover, this technology has been deployed for years to monitor pipeline infrastructures in the gas and oil sector, as well as other critical assets, including water networks [9]. Several studies have demonstrated leak detection in controlled or custom-built pipeline setups using distributed fiber optic measurements of temperature, strain, and vibration [10,11,12]. Nevertheless, transferring these concepts to urban water distribution systems is often limited by the need for dedicated fiber installations close to the pipe or even wrapping specialized fibers around pipelines [10,13,14,15]. As a consequence, the required in-field installation commonly involves substantial civil works (e.g., re-factoring and trenching), leading to high deployment costs for water network administrations.

In this paper, we demonstrate a system that leverages distributed acoustic sensing (DAS) to detect water leakage in urban environments. In contrast to dedicated sensing deployments, our approach takes advantage of the dense network of optical fibers already installed by telecommunication operators and public administrations in the vicinity of water pipelines [16]. As a consequence, spare dark fiber links can be used as water leak detection sensors, avoiding the high cost of fiber installation. The experimental field trial utilized the city’s existing fiber optic telecommunication networks to continuously monitor water pipelines. We developed several automatic leak detection algorithms to identify water leaks by analyzing the spectral characteristics of vibration signals acquired with a long-range, high-sensitivity DAS setup based on an optical pulse compression (OPC) scheme. These algorithms were developed following two approaches: (i) using conventional signal processing techniques that exploit specific features of the vibration signals and typically require prior knowledge of the expected vibration signatures and (ii) using sparse autoencoders that perform an automatic calibration under normal operating conditions and subsequently detect anomalies with respect to that baseline behavior. Both approaches take advantage of the fact that water leaks typically generate relatively high-frequency vibrations in a quasi-continuous regime, so time-averaging and spectrum-based analysis can be applied to reveal the associated spectral features.

## 2. Description of the Water Leakage Detection System Based on DAS

**Figure 1:**
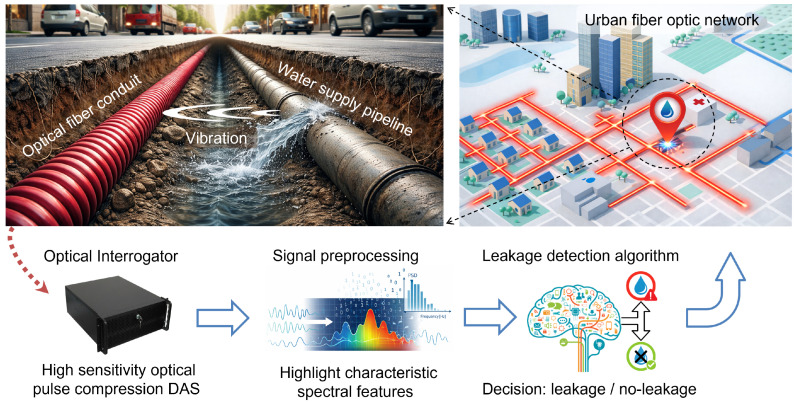
Schematic representation of the proposed urban water leakage detection and localization system.

The concept behind our automatic water leakage detection system is illustrated in Figure 1. The system capitalizes on the fact that urban water pipelines are typically buried underground near dense networks of fiber optic conduits that telecommunication operators and public institutions install for communication purposes. Water leaks generate acoustic vibrations that propagate through the ground and underground infrastructure, potentially reaching nearby fiber optic conduits. These vibrations are then detected by DAS sensor interrogators connected to dark fibers within the existing conduits. Alternatively, the DAS signal can be wavelength-multiplexed onto active communication fibers [17]. However, since water and communication networks are typically installed in separate tunnels or ducts, detecting the small vibrations induced by leaks is challenging. To address this, we propose using a DAS sensor based on optical pulse compression (OPC) implemented in a coherent optical time-domain reflectometry (COTDR) setup. This is the DAS sensor type that has the best capability to provide the longest possible range measurements in a purely passive link without additional distributed amplification [18]. This configuration launches long-duration, high-energy pulses with a high time bandwidth product into the fiber. Upon reception, these signals are processed using matched filters to achieve high-spatial-resolution effective pulse widths, significantly enhancing sensitivity. Combined with phase noise compensation, this approach enables measurement ranges of over 100 km [19]. Additionally, the DAS sensor measures differential phase shifts that are predominantly linear with the local vibrations induced by leaks at each sensing position along the fiber. This near-linear relationship provides a solid basis for robust post-processing strategies since the measured signal follows the vibration amplitude with minimal distortion and can therefore be mapped consistently into quantitative leakage detection metrics.

However, water leakages generate vibration signatures that are not readily discernible in time domain waveforms, which typically appear as non-stationary random fluctuations, often dominated by background activity. In contrast, frequency domain representations reveal characteristic energy distributions and high-frequency resonance peaks that provide a clearer separation between normal-condition vibrations and leakage-induced activity [20]. Therefore, the goal of the preprocessing block shown in Figure 1 is to convert the raw DAS traces into appropriate spectral representations that can be fed to the final stage of the system, where automatic leakage detection algorithms are implemented.

The output of the leakage detection algorithms is a scalar decision variable (or metric) that is used to flag the leakage/no-leakage condition at each sensing location. Different algorithmic strategies can be adopted to generate this decision variable, provided that it yields a robust separation between normal-condition and leakage-induced activity and can be thresholded to support a reliable binary decision. In this stage, rather than classifying individual samples, decisions are derived from the statistical properties of the detection metric distribution computed over a set of measurements within a given observation window. This strategy leverages the fact that leakage-induced vibrations are persistently present, whereas other environmental vibrations occur only sporadically and mainly contribute to the tails of the detection metric distribution, without significantly altering its mean behavior. In a practical implementation, distribution-based descriptors (e.g., the mean value of the detection metric) could be compared against the corresponding reference values obtained from the leakage-free training data. A leakage alarm would then be triggered when the selected statistic deviates beyond a predefined threshold. The detection metric is computed independently at each sensing position; therefore, the estimated leakage location could be obtained directly from the spatial distribution of the detection metric statistic along the fiber.

## 3. Field Demonstration on a Real Urban Environment

**Figure 2:**
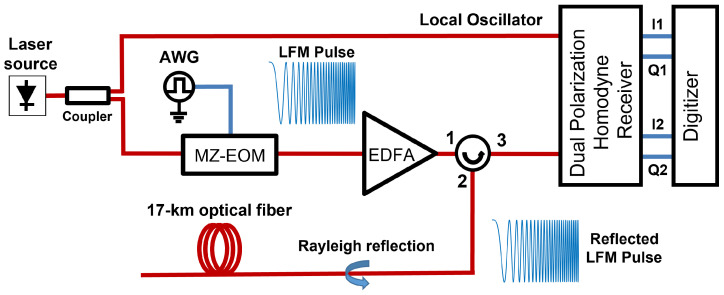
Experimental setup of the OPC-DAS interrogator.

The field measurements were performed with a benchtop OPC-DAS interrogator, whose experimental setup is shown in Figure 2. The output of a narrow-linewidth laser (NKT Koheras E15) operating at 1550 nm with a nominal linewidth of 100 Hz is split into two branches. One branch is routed to the local-oscillator input of a homodyne receiver comprising a 90° dual-polarization optical hybrid and four balanced photodetectors, providing phase and polarization diversity. The second branch drives a Mach-Zehnder electrooptic modulator (MZ-EOM), which transfers to the optical domain the linear-frequency-modulated (LFM) pulses generated by an arbitrary waveform generator (AWG). The MZ-EOM is biased at minimum transmission to produce double-sideband suppressed-carrier modulation, yielding two simultaneous LFM pulses symmetrically placed around the optical carrier. The modulated signal is amplified by an erbium-doped fiber amplifier (EDFA) and launched into the sensing fiber. The backscattered light is then detected by the homodyne receiver, digitized, and post-processed on a computer. The digitized waveform goes through a pulse-compression stage implemented by digitally cross-correlating it with the ideal LFM reference. Fading suppression is implemented by combining the rotated vector sum method with a spatial moving average [21]. Then, the phase difference between positions separated by the gauge length is computed independently for each sideband. Finally, these differential-phase signals, which constitute frequency–diversity branches with different fading statistics, are combined for each polarization and then vectorially summed across polarizations [22].

The urban water supply network of the city of Pamplona (approximately 200,000 inhabitants) was selected to demonstrate the proposed water leakage detection system. The experiments were conducted using a spare dark fiber within a 17 km telecommunication cable currently used by the Municipality of Pamplona to connect several of its premises. To simulate the water leaks, we had the invaluable assistance of the public company managing the water supply infrastructure of the Municipality of Pamplona, which has dedicated technicians for detecting water leaks in its network. Water leakages were simulated by activating a drainage valve installed on a 400 mm diameter asbestos-cement pipe, which is part of the water supply network. The leakage flow was controlled by adjusting the valve opening. Although this approach did not reproduce all the hydraulic and mechanical details of a natural leakage site, it provided a practical and controlled way to generate the leak-induced vibrations of interest in this work. Real leak-induced vibrations can be highly heterogeneous since they depend on the specific defect geometry and the hydraulic and environmental conditions of each case. Therefore, no controlled emulation can reproduce the full variability of real leakage scenarios. In our case, the valve-induced signals exhibited spectral features comparable to those observed in real leak inspections using a commercial punctual electro-acoustic water leakage detector (Sewerin Aquaphone A200) routinely employed by the local water utility maintenance staff. When the drainage valve was activated, the leak-induced vibrations propagated through the surrounding soil and reached the nearby dark fiber link described above, which was installed in a separate trench running parallel to the water supply pipeline, at an approximate lateral separation of 1 m. These vibrations were then captured with the OPC-DAS interrogator located approximately 2.97 km from the leakage site.

**Figure 3:**
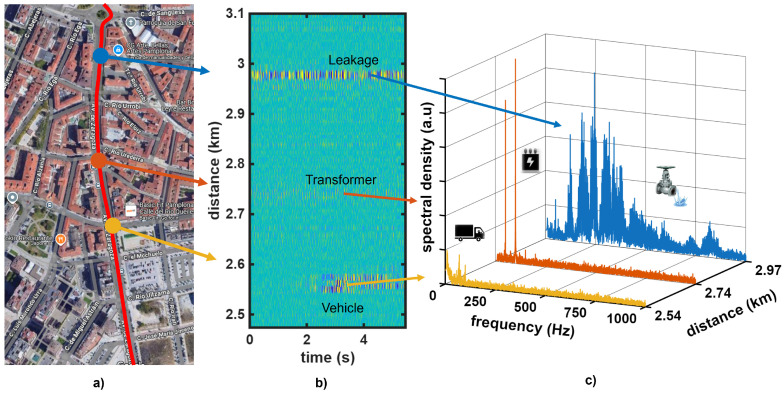
Field example of vibration events captured by the OPC-DAS sensor along the urban dark fiber route. (**a**) Map view of the interrogated fiber path (red line) and three annotated locations corresponding to the leakage location (blue marker), an electrical transformer (orange marker), and a road intersection (yellow marker) (source: Google Maps [23]). (**b**) Waterfall representation of the measured strain for a 600 m fiber section over a 5 s interval, showing the vibration induced by a controlled leakage of 3.75 L/s, together with additional activity at other fiber locations. (**c**) Corresponding spectra extracted at the annotated locations.

Figure 3a shows a map view of a 600 m section of the interrogated fiber (red line), with the leakage location indicated by the blue marker. Figure 3b presents the corresponding waterfall representation of an example strain measurement performed with the OPC-DAS over a 5 s interval while a leakage of 3.6 L/s was generated. Vibrations around the valve location are clearly visible. As shown in Figure 3c, the spectrum of the detected vibration exhibits a rich combination of frequency components extending to high frequencies. Additionally, vibrations were detected at other locations along the fiber section. For example, the location marked with the orange arrow shows a vibration that was consistently present. Spectral analysis revealed sharp peaks at multiples of 50 Hz, suggesting electrical equipment such as a transformer. Its effect remained highly localized, and no significant spreading to neighboring sensing positions was observed. This vibration was only observed during the night, which indicated that it was likely associated with the street-lighting infrastructure at that location. In contrast, the short-duration vibrations marked with the yellow arrow were caused by passing vehicles near a crossroads. Notice that these vehicle-induced vibrations were intermittent since traffic was not continuous, and some time intervals contained no events. This temporal sparsity enabled a statistical treatment of the acquired samples, so that persistent leakage-induced vibrations could be highlighted with respect to transient disturbances. In the measurements considered in this work, most sensing positions exhibited a relatively flat vibration spectrum above a few hertz, as persistent vibration patterns were rarely encountered in practice.

To analyze the vibration signatures associated with different leakage conditions, LFM pulses with a 7 μs duration and a 380 MHz bandwidth centered at 200 MHz were used, resulting in a spatial resolution after matched filtering of 0.26 m. The pulse duration was optimized for the measurement distance to avoid the deleterious effect of laser phase noise in the measurement [24]. A gauge length and a spatial moving average of 2.6 m were used to mitigate fading. The pulse repetition frequency was set to 2 kHz, close to the time-of-flight limit imposed by the 17 km fiber. Therefore, the effective measurable acoustic bandwidth in these experiments was approximately 1 kHz, which was sufficient to capture the main spectral components of the leak-induced vibrations. Measurements were conducted during the night, replicating the working procedures of the technicians, who operated in night shifts to minimize the impact of environmental vibrations caused by traffic, pedestrians, or other noise sources. To enable automatic and continuous monitoring of the water supply network, a calibration process was required to record the vibrations at each position along the fiber under normal-condition operation. For this purpose, 300 acquisitions of 5 s were collected at random intervals throughout the night in the absence of leakage. Then, eight leakage flow settings were investigated, ranging from 0.14 to 3.75 L/s. For the 280 mL/s, 480 mL/s, and 680 mL/s cases, 60 acquisitions of 5 s were recorded per setting, whereas 12 acquisitions of 5 s were recorded for the remaining settings. Table 1 summarizes the dataset used in the experiments.

**Figure 4:**
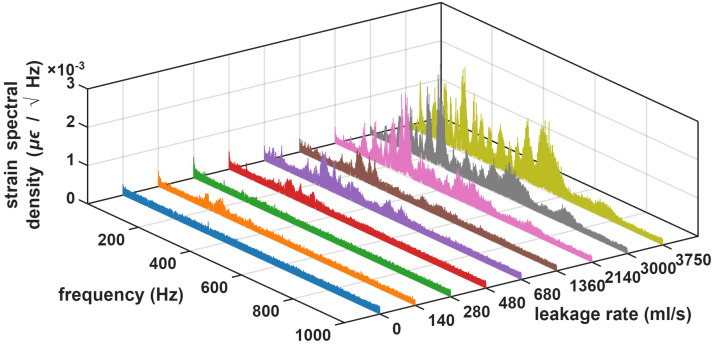
Spectra of the measured vibration signals for each leakage rate at the position of the valve.

Figure 4 depicts the spectra of the strain measurements acquired by the OPC-DAS interrogator at the valve location for all records, including no-leakage cases. Overall, as leakage increased, both spectral amplitude and effective bandwidth tended to rise, although the trend was not strictly monotonic. Some records exhibited low-frequency components, which could be attributed to ambient vibrations during normal operation, mainly road traffic and pedestrians. The non-leakage condition (blue traces) exhibited an approximately flat spectrum with low amplitudes over the analyzed band. Variability across records was in part due to changes in the sensor’s sensitivity associated with Rayleigh fading. For the first leakage setting (140 mL/s, orange trace), the spectrum changed markedly, with a clear amplitude increase and dominant peaks in the 150–200 Hz range. However, for the next setting, where the leakage flow was increased to 280 mL/s (green traces), the vibration level decreased and the spectrum exhibited smaller peaks. This is consistent with the fact that leakage-induced vibrations are inherently stochastic and not strictly proportional to leakage flow, even though a clear overall tendency was observed in our measurements. For the third setting (480 mL/s, red traces), the average spectral level increased again and multiple peaks appeared between 200 and 400 Hz. For leakage flows between 680 and 1360 mL/s, pronounced components were observed around 500 Hz and, for the highest tested flows, (2140–3750 mL/s), additional weaker peaks emerged near 800 Hz.

## 4. Automatic Water Leakage Detection Algorithms

**Figure 5:**
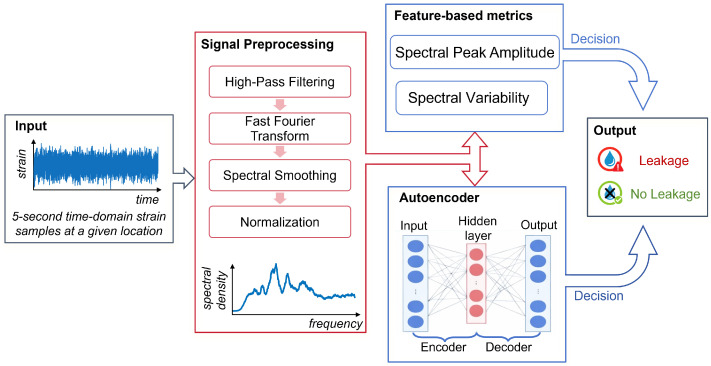
Automatic urban water leakage detection workflow.

**Figure 6:**
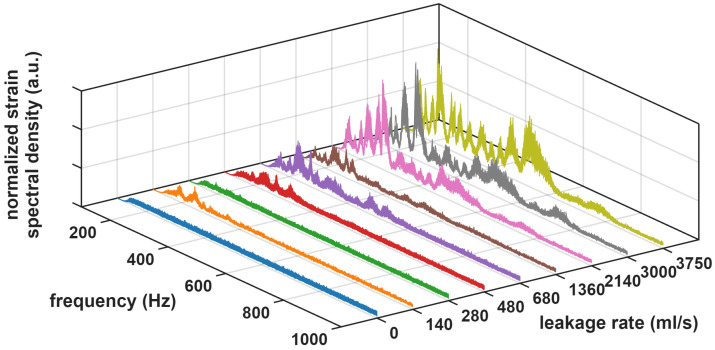
Spectra of the measured vibration signals for each leakage rate at the position of the valve after the preprocessing stage.

Once the vibrations coupled to the sensing fiber were captured by the OPC-based DAS interrogator, several automatic leakage detection algorithms were implemented. The overall workflow is summarized in Figure 5. The preprocessing block, highlighted in red in Figure 5, processed 5 s time-domain strain traces that were extracted and handled independently for each sensing location along the fiber. As a first step, the time-domain traces were passed through a high-pass filter to suppress low-frequency components that may have been contaminated by ambient vibrations induced by road traffic, pedestrians, and other normal-condition activity on the pavement. This also reduced the impact of slow perturbations, such as temperature-driven refractive-index variations, thermal expansion, or quasi-static microbending, which were expected to evolve predominantly at low frequencies. Specifically, a 150 Hz high-pass filter was applied with a passband ripple below 1 dB. The filtered traces were then transformed into the frequency domain using Fast Fourier Transform (FFT). Finally, the filtered spectra were smoothed using a 10 Hz moving-average filter and normalized to equalize the noise level across acquisitions. This improved comparability across time intervals and operating conditions and avoided amplitude-driven bias during model training. The spectra after preprocessing are shown in Figure 6. They preserved the same overall tendencies observed in the raw data of Figure 4, but with reduced noise-induced variability, yielding more consistent spectral patterns across measurements.

In the final decision stage, two independent strategies were considered (blue boxes in Figure 5). First, a feature-based approach computed spectral metrics from the measured spectra to obtain scalar indicators for leakage/no-leakage decisions. Second, we developed an unsupervised method based on sparse autoencoders trained using only no-leakage data.

**Figure 7:**
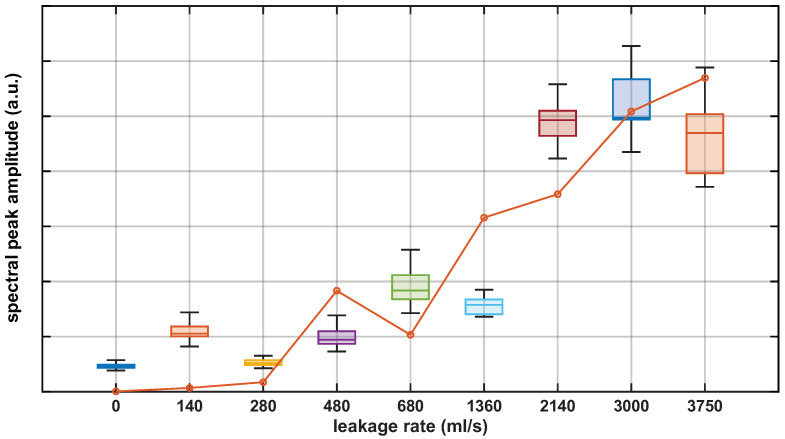
Spectral peak amplitude metric for the preprocessed DAS measurements (boxplots) and electro-acoustic punctual sensor (orange line).

The first feature-based metric that we propose is motivated by the observation that, as shown in Figure 6, under no-leakage conditions, the spectra of the preprocessed vibration measurements did not exhibit dominant resonance peaks. In contrast, leakage conditions produced pronounced peaks within specific frequency bands, which could be exploited as a leakage indicator. Accordingly, we defined a spectral peak amplitude metric by applying a peak detection procedure to the normalized spectra. For each measurement, the corresponding spectrum was scanned to identify its local maxima, and the metric was defined as the amplitude of the most prominent spectral peak, i.e., the local maximum with the highest amplitude. Figure 7 reports the resulting metric for the preprocessed measurements at the valve location. The boxplots in Figure 7 summarize each dataset using the median (central line) and interquartile range (IQR, 25th–75th percentiles). Whiskers extend to the most extreme data points within 1.5×IQR, while values outside this range are treated as outliers. All subsequent figures that include boxplots follow the same definition and graphical conventions. Overall, the results show that this metric enabled automatic detection for all leakage flow rates tested at this location. In addition, these results were compared against the commercial punctual electro-acoustic water leakage detector by performing a set of measurements with the same leakage rates and computing the spectral peak amplitude metric. The results are depicted in Figure 7 (orange trace), showing a strong correlation with the DAS-based metric. Minor deviations can be partly explained by small differences in the effective leakage condition between measurement days, and mainly by the different sensing mechanisms and coupling points, since the optical fiber was buried, while the Aquaphone transducer was manually coupled to the pavement surface.

**Figure 8:**
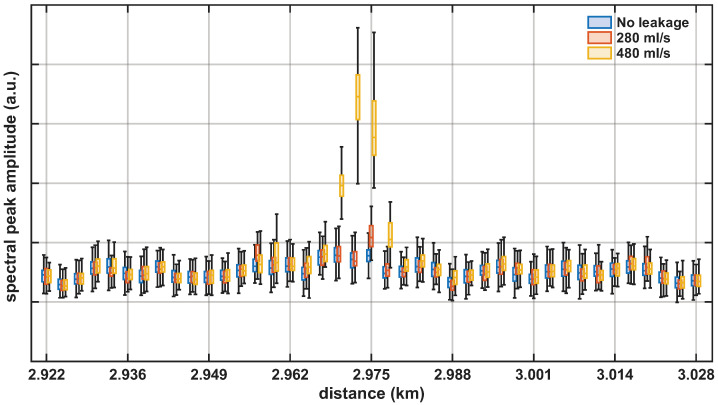
Spectral peak amplitude metric over a 105 m span centered at the valve location: boxplots for no leakage (blue), 280 mL/s (orange), and 480 mL/s (yellow).

Figure 8 shows the spectral peak amplitude metric over a 105 m span centered at the valve location, where the leak was generated. The cases of 280 mL/s (orange boxplots) and 480 mL/s (yellow boxplots) are reported because they correspond to the weakest leakage-induced vibrations among the tested conditions. The no-leakage condition (blue boxplots) remained at a low level across the span, although some variability was observed. As a result, the 280 mL/s case was only marginally separable, even though its boxplot at the valve location yielded the highest values in the span. For 480 mL/s, high values also extend to adjacent positions, indicating that the leakage-induced vibrations spread spatially along the pipeline. For higher leakage rates, a broader spatial response was observed, as more vibration energy was coupled to the surrounding medium. Overall, this facilitated detection, although, in some cases, it may have reduced localization precision because the spatial spreading was not necessarily symmetric around the leakage position.

**Figure 9:**
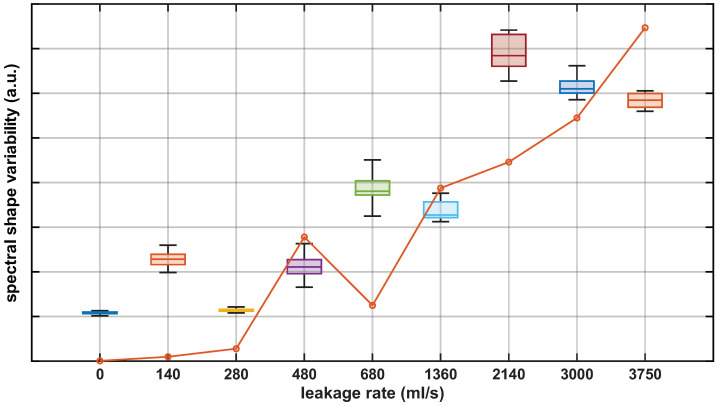
Spectral shape variability metric for the preprocessed DAS measurements (boxplots) and the electro-acoustic punctual sensor (orange line).

As a second feature-based metric, we considered spectral variability analysis for leakage detection. Under normal operating conditions and in the absence of a persistent vibration source, the spectrum was approximately flat and exhibited low variability levels. In contrast, leakage conditions typically increased spectral variability. To quantify this variability, the spectrum was partitioned into 100 segments and the mean value of each segment was computed. The spectral shape variability metric was then defined as the coefficient of variation of these segment means, i.e., the standard deviation normalized by their arithmetic mean. Figure 9 shows the spectral shape variability metric computed from the preprocessed measurements at the valve location. The observed trends are consistent with those obtained using the spectral peak amplitude metric in Figure 7. The same metric computed from the punctual electro-acoustic water leakage detector measurements (orange line in Figure 9) showed consistent results, in line with the behavior observed for the previous metric.

**Figure 10:**
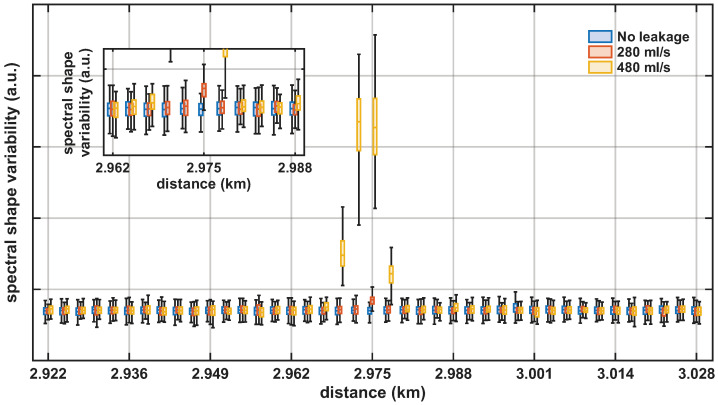
Spectral shape variability metric over a 105 m span centered at the valve location: boxplots for no leakage (blue), 280 mL/s (orange), and 480 mL/s (yellow). Inset: zoom around the leakage location highlighting the localized spectral shape variability increase for the 280 mL/s leakage flow.

Figure 10 shows the spectral shape variability metric for the 280 mL/s and 480 mL/s leakage cases over a 105 m span centered at the valve location. The inset in the figure depitcs a zoomed view around the leakage location. The no-leakage condition remained at a consistently low level across the span. In contrast, both leakage cases exhibited a clear increase of the metric at the leakage location, enabling discrimination from the no-leakage baseline. Compared with the spectral peak amplitude metric, this feature exhibited lower variability under no-leakage conditions and provided clearer detectability at the valve location. For 480 mL/s, high spectral shape variability values also extended to neighboring locations, indicating spatial spreading of the leakage-induced vibrations.

Feature-based metrics such as those described above are derived from prior knowledge of the signal characteristics. Consequently, human intervention is required to define the features and tune the associated decision rules. Moreover, the background vibration environment varies along the fiber, so the metric often has to be adapted to each sensing location, with different levels of manual effort. In addition, their performance may degrade in the presence of persistent non-leakage vibration sources, with broadband spectral content overlapping that of leak-induced signals. To address this limitation, we propose an unsupervised learning approach for leakage detection and localization. The key idea is to use autoencoders to learn a baseline representation from data acquired under leakage-free operating conditions and identify leaks by detecting deviations from this baseline in the spectral characteristics of the measured signals. Unlike the previous feature-based metrics, an autoencoder does not require a flat background spectrum since it learns the normal vibration pattern at each sensing location from the available no-leakage data and detects deviations from that learned baseline. Because the leakage-free vibration patterns are location-dependent, a location-specific autoencoder is required (i.e., one model per sensing position).

In our implementation, we adopted sparse autoencoders (SAEs), i.e., unsupervised neural networks that learn sparse latent representations by reconstructing the input under a sparsity constraint on the hidden activations [25]. SAE-based reconstruction models have been successfully used for industrial anomaly and fault detection when only normal data are available for training [26]. Unlike supervised classifiers, an SAE does not require labeled leak examples and is trained in a self-supervised manner by minimizing reconstruction loss on leakage-free data. In its simplest form, an SAE consists of an input layer, a latent (hidden) layer, and an output layer. The latent layer constrains the information flow and, together with sparsity regularization, forms an effective bottleneck that encourages a compact representation.

Table 2 summarizes the architecture and training hyperparameters of the proposed SAE. The SAE adopts a fully connected architecture with a single hidden layer, composed of one encoder layer and one decoder layer. The input to the network is the preprocessed spectrum obtained from each measurement sample, and the output layer has the same dimension so that the input can be reconstructed. In our configuration, the input and output layers had 2500 units, whereas the hidden layer comprised 300 neurons. To improve feature selectivity, sparsity constraints limited the average activation of hidden units so that only a small subset was active for a given input. We combined an $L_{2}$ weight-decay term ($\lambda =10^{-4}$) with a sparsity penalty (β=0.05) and a target average activation of ρ=0.2, and training was performed by minimizing the resulting reconstruction loss with regularization. This combination mitigates overfitting and promotes stable generalization across operating conditions. Owing to its shallow, fully connected architecture, the SAE comprised approximately $1.5\times 10^{6}$ trainable parameters, which provided a quantitative indication of its model size and computational requirements.

Structurally, the encoder applied a nonlinear activation to map the input into the interval (0,1):

(1) $$h=\mathrm{logsig}\;\left(Wx+b\right),$$

where *x* is the input vector, *W* is the weight matrix, and *b* is the bias vector. The decoder used a linear transfer function to reconstruct the input:

(2) $$x^{\prime }=W^{\prime }h+b^{\prime },$$

where $W^{\prime }$ and $b^{\prime }$ denote the decoder parameters.

**Figure 11:**
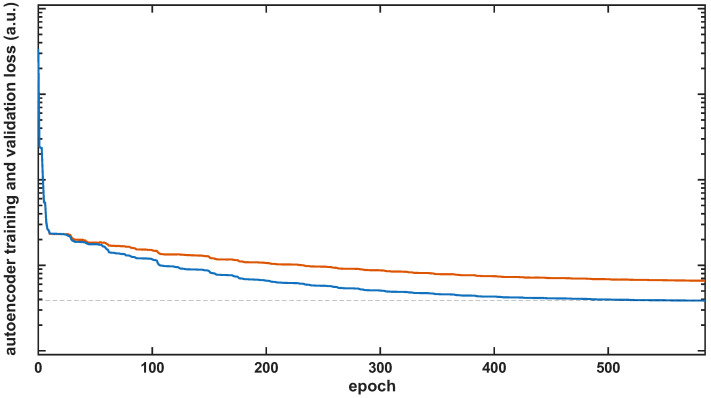
Training (blue) and validation (orange) reconstruction loss as a function of the epoch for a randomly selected sensing location.

Only no-leakage measurements were used for SAE model development, so the SAE learned a representative model of the leakage-free state and optimized its reconstruction performance for that condition. To evaluate the generalization ability of the model and reduce the risk of overfitting, the no-leakage dataset was randomly divided into training and validation subsets using an 8:2 ratio so that the validation data were not used for parameter fitting. Training was performed for a maximum of 1000 epochs. Automatic data scaling was disabled to preserve the original data distribution. Figure 11 shows the evolution of the training and validation loss for a representative sensing location during the SAE training process. Both curves decreased steadily during training and the validation loss remained slightly above the training loss while following the same overall trend. This behavior indicated that the model generalized consistently to unseen no-leakage data and did not exhibit significant overfitting. For this example, the minimum training loss, marked by a dashed line in the figure, was reached at approximately epoch 560.

**Figure 12:**
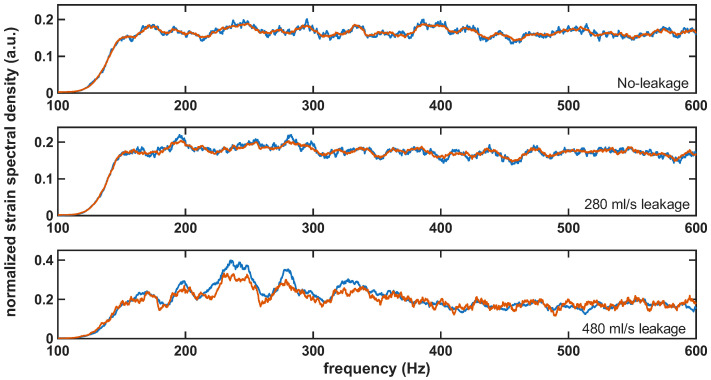
Representative SAE spectral reconstructions for normal-condition and leakage cases (280 mL/s and 480 mL/s), showing the original spectrum (blue) and the reconstructed spectrum (orange) in each subplot.

As with the previous feature-based metrics, anomalies were assessed during detection using a scalar indicator, which, in this case, was the reconstruction mean squared error (MSE) provided by the SAE. In the subsequent evaluation, this reconstruction MSE was computed for the held-out validation no-leakage data and the leakage measurements. Figure 12 shows representative reconstructions for normal-condition and leakage measurements. For normal-condition data, the reconstructed spectrum closely matched the input, indicating that the SAE accurately modeled the leakage-free baseline. For a leakage flow of 280 mL/s, small mismatches appeared mainly in the 200–300 Hz band. For 480 mL/s, the reconstruction error was substantially larger, consistent with a stronger deviation from the spectral characteristics learned under normal-condition operation.

**Figure 13:**
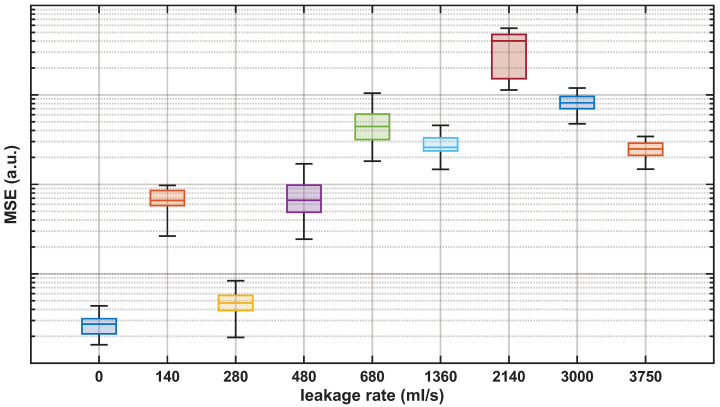
Boxplot representation of the MSE for the reconstructed DAS measurements after going through the autoencoder as a function of the leakage rate.

Figure 13 shows the distribution of the reconstruction MSE for the preprocessed measurements at the valve location after passing through the autoencoder. The results confirm that the autoencoder-based approach provided reliable leakage detection at the leakage location. All tested leakage conditions yielded reconstruction error distributions that were clearly shifted with respect to the no-leakage baseline, enabling robust separation between leakage and normal-condition operation. Overall, the evolution of the reconstruction error statistic across leakage settings followed the same qualitative behavior observed with the feature-based metrics, indicating that the SAE captured the underlying changes in the vibration spectra induced by leakage.

**Figure 14:**
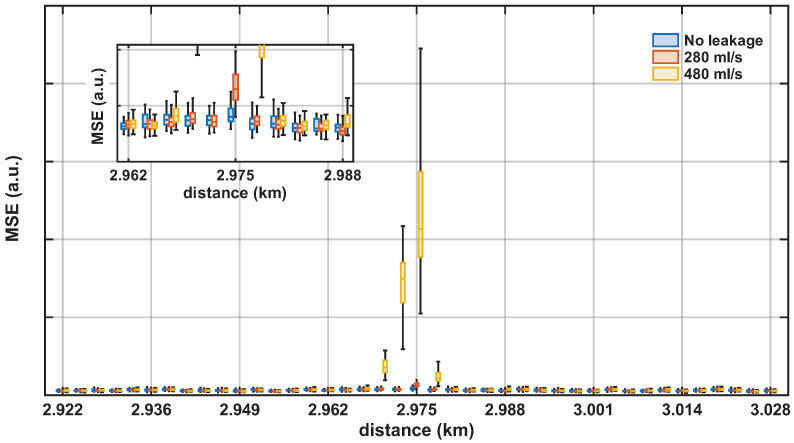
Reconstruction MSE after the SAE over a 105 m span centered at the valve location: boxplots for no leakage (blue), 280 mL/s (orange), and 480 mL/s (yellow). Inset: zoom around the leakage location highlighting the localized MSE increase for the 280 mL/s leakage flow.

Figure 14 illustrates the distribution of the reconstruction MSE after the autoencoder for the 280 mL/s and 480 mL/s leakage cases over a 105 m span centered at the valve location. The inset in the figure depitcs a zoomed view around the leakage location, showing that even for the 280 mL/s leakage, the reconstruction error was markedly higher than at the surrounding positions, indicating that the SAE identified anomalous spectral patterns at this point. For the 480 mL/s leakage case, the MSE exhibited a pronounced increase at the leakage position. In addition, adjacent positions also presented elevated MSE compared with normal-condition data, consistent with the spatial propagation of leakage-induced vibrations along the pipeline. For the remaining locations, MSE values remained in a low range, with only minor differences between leakage and normal-condition measurements. Overall, the localized maximum in reconstruction error at the leakage site, together with comparatively stable MSE at non-leak locations, supports the effectiveness of the proposed approach for spatial leakage localization. Compared with feature-engineered or model-driven approaches, the proposed method defines its detection metric directly from deviations with respect to a learned normal-condition baseline, rather than from assumptions about the specific spectral characteristics of leakage signals. This approach enables an automated framework that adapts to location-dependent signal characteristics and supports pipeline leakage monitoring. Therefore, although the SAE provides an adaptive anomaly detection framework, it is not specifically tuned to leakage detection, so any persistent change in the local vibration environment may also be flagged and should be regarded as a potential source of false positives.

## 5. Conclusions

We proposed and experimentally demonstrated a scalable urban water leakage monitoring system based on OPC-DAS operating over already deployed fiber links from existing telecommunication networks. The technique exploits the fact that time-averaging and spectrum-based analysis can be used to highlight leakage-related components while suppressing transient disturbances. Several leakage detection algorithms were developed and experimentally validated using a real field scenario. First, we introduced two lightweight feature-based metrics derived from the vibration spectra. Second, we implemented an autoencoder-based detection strategy to enable blind leakage identification without requiring prior knowledge of the spectral patterns produced by leaks. By learning the normal-condition behavior at each sensing position and flagging deviations from that baseline, the autoencoder approach provides an adaptive mechanism that is less dependent on handcrafted assumptions.

In a practical implementation, the sensing range could be extended by sequentially switching a single DAS interrogator among multiple fiber routes deployed across the city at, for example, fiber distribution hubs, thereby extending coverage without requiring a dedicated interrogator per link. However, the performance of the proposed system is inherently location-dependent, as the mechanical coupling between the pipe, the soil, and the communication conduit is highly site-dependent. In addition, since the autoencoder acts as an anomaly detector with respect to a learned baseline, the method can be less effective in highly noisy environments where persistent high-frequency vibro-acoustic emissions are continuously present or when the background vibration pattern changes after training due to non-leakage causes. Despite these constraints, the results indicate that the technique can be an effective tool for early warning and precise localization of leaks in urban environments. Future work should evaluate the proposed technique under more challenging field conditions, including locations with persistent broadband non-leakage vibration sources and naturally occurring real leakages, in order to assess its robustness in harsher urban environments.

## Figures and Tables

**Table 1:** Summary of the dataset used for leakage detection experiments. Each sample consisted of one preprocessed vibration spectrum extracted at a given sensing location from a single 5 s acquisition record.

Condition	Number of Samples
No leakage	300
140 mL/s	12
280 mL/s	60
480 mL/s	60
680 mL/s	60
1360 mL/s	12
2140 mL/s	12
3000 mL/s	12
3750 mL/s	12
Total	540

**Table 2:** Architecture and training configuration of the sparse autoencoder used for automatic water leakage detection.

Parameter	Value
Architecture	Sparse autoencoder
Input size	2500
Output size	2500
Number of hidden layers	1
Hidden layer size	300 neurons
Encoder activation	Sigmoid
Decoder activation	Linear
Loss function	Reconstruction MSE with regularization
Training algorithm	Scaled conjugate gradient
$L_{2}$ weight regularization, λ	$10^{-4}$
Sparsity regularization, β	0.05
Sparsity target, ρ	0.2
Maximum number of epochs	1000
Training subset	80% of the no-leakage data
Validation subset	20% of the no-leakage data

## Data Availability

The data presented in this study are available on reasonable request from the corresponding author.

## Abbreviations

The following abbreviations are used in this manuscript:

AEI	Agencia Estatal de Investigación (Spain)
ANIMSA	ANIMSA S.A.U. (company name)
AWG	arbitrary waveform generator
COTDR	coherent optical time-domain reflectometry
CSC	China Scholarship Council
DAS	distributed acoustic sensing
EDFA	erbium-doped fiber amplifier
ERDF	European Regional Development Fund
FFT	Fast Fourier Transform
IQR	interquartile range
LFM	linear frequency-modulated
MCIN	Ministerio de Ciencia e Innovación (Spain)
MSE	mean squared error
MZ-EOM	Mach–Zehnder electrooptic modulator
OPC	optical pulse compression
OPC-DAS	optical pulse compression distributed acoustic sensing
SAE	sparse autoencoder
SNR	signal-to-noise ratio
